# Determination of the Expression of PD-L1 in the Morphologic Spectrum of Renal Cell Carcinoma

**DOI:** 10.7150/jca.35738

**Published:** 2020-03-26

**Authors:** Beatriz Walter, Sara Gil, Xu Naizhen, Michael J Kruhlak, W Marston Linehan, Ramaprasad Srinivasan, Maria J Merino

**Affiliations:** 1 Translational Surgical Pathology Section, Laboratory of Pathology, National Cancer Institute, National Institutes of Health, Bethesda, Maryland, 20892, USA.; 2 Experimental Immunology Branch, National Cancer Institute, National Institutes of Health, Bethesda, Maryland, 20892, USA.; 3 Urologic Oncology Branch, National Cancer Institute, National Institutes of Health, Bethesda, Maryland, 20892, USA.

**Keywords:** PD-L1 expression, renal cell carcinoma, RCC histological subtypes, HLRCC

## Abstract

Immunotherapy is reportedly an effective form of therapy for some advanced cancers such as lung adenocarcinoma, malignant melanoma and colorectal adenocarcinoma. In renal cell carcinoma (RCC), the role of immunotherapy is under investigation. Programmed Death-Ligand 1 (PD-L1) is a molecule expressed on the surface of certain tumor cells and binds to the Programmed cell death protein 1 (PD-1) on cytotoxic T-cells, an interaction that inhibits the antitumor immune response. The aim of this study is to evaluate PD-L1 expression in the morphologic spectrum of RCC. A total of 172 cases of RCC comprising all types were studied and the PD-L1 was correlated with immune response for CD4 and CD8. Positive membranous staining for PD-L1 was seen in 59 (34%) of the 172 samples. The positive cases were HLRCC (31/53), Type 1 Papillary RCC (10/31), Chromophobe (7/20), Hybrid (3/9), TFE-3 related cancer (3/8), Undifferentiated (3/5), and TFEB tumors (2/2).

Clear cell carcinomas, Oncocytomas and SDHB deficient-RCC didn't show any expression of PD-L1; (0/34;0/7;0/3). Our results demonstrated that aggressive forms of RCC such as HLRCC have high expression of PD-L1, in contrast to clear cell renal carcinomas. Our findings support a possible role of anti-PD-L1/PD-1 immunotherapies in the treatment of PD-L1-positive RCC.

## Introduction

Kidney cancer accounts for approximately 4% of all neoplasms reported annually in the United States. It is estimated that there will be 403,260 new cases in 2018 with 175,098 deaths worldwide 1-3. Renal cell carcinoma (RCC) includes a broad spectrum of kidney morphologies that may have an indolent or very aggressive clinical behavior 2.

RCC is frequently resistant to conventional forms of therapies. However, over the past decade, a variety of 'targeted' agents have proven effectiveness in RCC and have received regulatory approval by the U.S Food and Drug Administration (USFDA) for patients with advanced cancers 4. Inhibitors of the VEGF pathway, such as sunitinib and pazopanib, are often recommended for patients with metastatic RCC. However, these treatments are seldom curative, since most patients eventually progress and die from their cancers. Recently, agents targeting the PD1/PD-L1 pathway have demonstrated efficacy as single agents and in combination with other therapies and may eventually play a prominent role in the management of patients with kidney cancer 4-6.

The programmed cell death-1 (PD-1)/PD1 ligand (PD-L1) pathway is an important checkpoint for regulation of T cell-mediated immune responses 7. It consists of the transmembrane protein PD-1/CD279 itself and its 2 ligands PD-L1 (B7-H1, CD274) and PD-L2 (B7-DC, CD273). These PD-Ls activate PD-1, which results in a reversible inhibition of T-cell activity and proliferation, also known as T-cell exhaustion or anergy 8. Unfortunately, malignancies can also use these immunosuppressive effects of the PD-1/PD-L pathway 9, which is reflected by high levels of PD1-positive tumor infiltrating T- cells. Several tumors such as lung adenocarcinoma and breast cancers are known to express PD-L1 as one of the mechanisms of building a defense line against tumor-infiltrating lymphocytes (TILs) 10.

Inhibition of the PD-1/PD-L1 pathway enhances antitumor immunity preventing tumor cells from escaping from host T-cell responses, providing a new strategy for tumor immunotherapy 11. Recently promising results of PD-1/PD-L1 blockade have been reported in Hodgkin lymphomas, melanomas, and non-small cell lung cancers 12, 13.

Under normal conditions, PD-1 is expressed on activated CD8^+^ T cells. Its interaction with PD-L1 on host tissues leads to the inhibition of TCR (T Cell Receptor) signaling, limiting the interaction between T cells and target cells, and ultimately leading to T-cell inactivation. PD-L1 expression can be induced by inflammatory stimuli, such as interferons, which are released by tumor infiltrating lymphocytes. The PD-L1 induction process has been termed “adaptive immune resistance” 13. It represents a mechanism by which cancer cells protect themselves from immune-cell mediated tumor cell killing. This findings led to the clinical development of antibodies blocking PD-1 or PD-L1, resulting in clinical responses in a variety of malignancies; such as melanomas, non-small cell lung carcinomas, and diffuse large B-cell lymphoma 11, 13. The question of whether or not the evaluation of the expression of PD-1 and PD-L1 by immunohistochemistry may be significantly useful during the treatment of RCC has not yet been answered. In this study, we used immunohistochemistry (IHC) and multiplex IF (mxIF) to evaluate the role of PD-L1 expression in patients with surgically resected RCC. We examined the association between the expression of PD-L1, PD-1, CD4 and CD8 and various clinicopathological characteristics, histological subtypes, and extent of metastasis (TNM).

## Materials and Methods

A total of 172 cases encompassing different RCC subtypes were obtained from the surgical pathology archives of the Laboratory of Pathology, National Cancer Institute, Bethesda, MD, USA under an IRB approved protocol. Hematoxylin and eosin (H&E) stained slides were reviewed to confirm the cancer diagnosis, and to further classify the morphologic type. Medical records were reviewed, and available clinical data was obtained (see Table 1). These tumors included 53 with Hereditary Leiomyomatosis and Renal Cell Cancer (HLRCC), 34 Clear Cell Renal Cell Carcinoma (CCRCC with VHL), 31 Type 1 Papillary Renal Cell Carcinoma (Type 1 PRCC), 20 Chromophobe Renal Cell Carcinoma, 9 Hybrid Tumors, 8 Renal Cell Carcinoma associated with Xp11.2 translocation and transcription Factor E3 expression (RCC- TFE3), 7 Renal Oncocytomas, 5 Undifferentiated Renal Cell Cancers, 3 RCC with germline succinate dehydrogenase B mutation (SDHB) deficient, and 2 tumors with t (6; 11) translocation or TFEB-amplified (TFEB-RCC) (see Table 2).

### Immunohistochemistry (IHC)

For each case, available slides of the tumor were reviewed and selected for especial studies, as PD-L1, PD-1, CD4 and CD8 stains.

### PD-L1 and PD-1 staining

Immunohistochemical (IHC) staining was preceded by antigen retrieval (20 minutes), achieved by steaming deparaffinized and rehydrated sections in Tris-EDTA, pH 9. After protein blocking, the sections were incubated with a primary antibody rabbit anti PD-L1 clone E1L3N (Cell Signaling. Danvers, MA) 1:50 dilution overnight at 4° C. Antibody binding was detected using HRP-polymer and visualized with 3,3'- diaminobenzidine (DAB) (DAKO EnVision™+ System, HRP). The positive control used for PD-L1 IHC was human mature placenta, and MCF-7 cell line FFPE block for negative PD-L1 protein expression.

For PD-1, we used a primary anti mouse PD-1 antibody, clone EH33 (Cell Signaling. Danvers, MA) at 1: 100 dilutions, following the same procedure described above, and using lymph node as a positive control.

**Figure 1:**
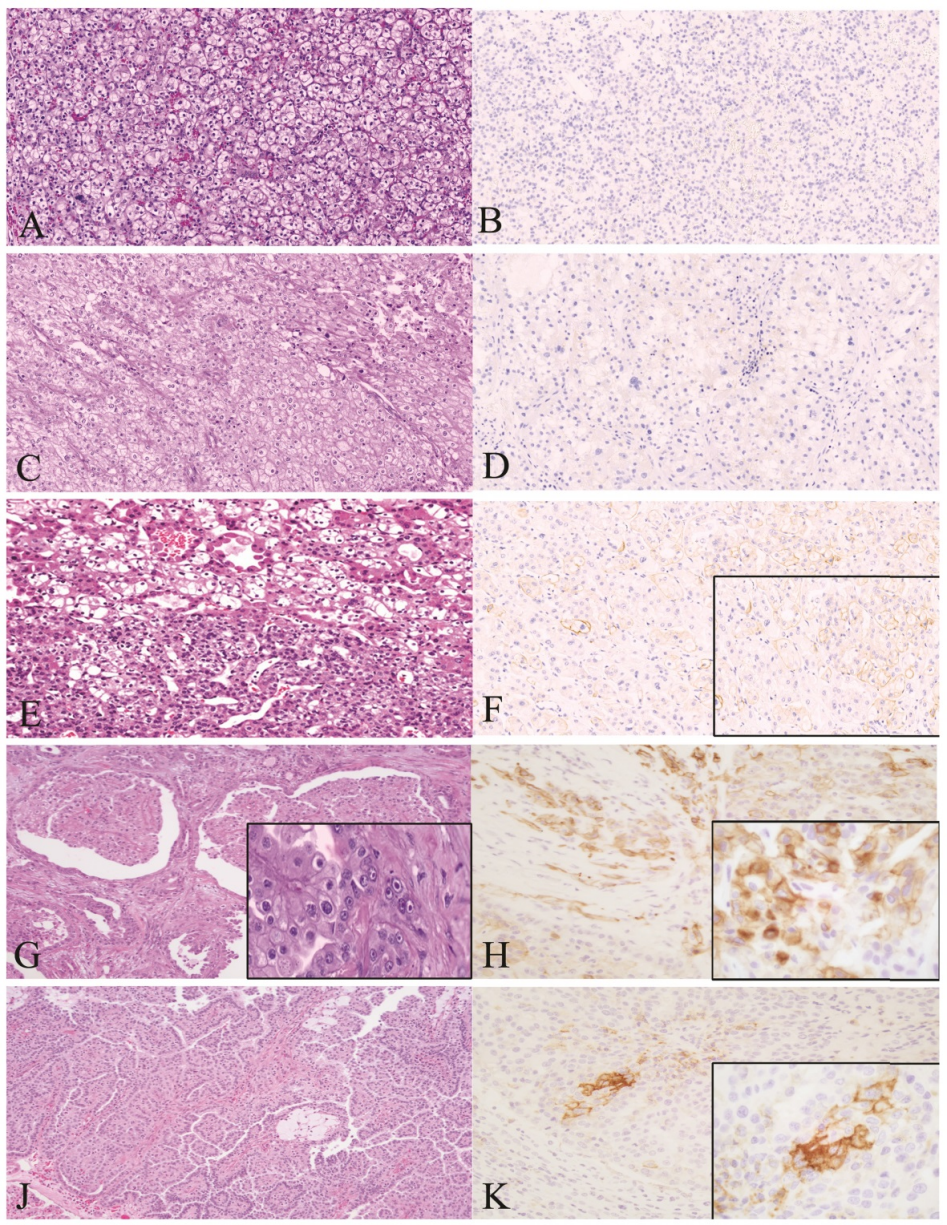
Representative histopathological images of malignant tumors stained for H&E and PD-L1 by IHC (membranous brown color). Left: H&E staining. Right: PD-L1 positive cells. A1, A2: Clear Cell RCC. B1, B2: RCC Chromophobe type. C1, C2: HLRCC. D1, D2: RCC papillary type 1. Magnification 40x. RCC: Renal Cell Carcinoma.

The immunohistochemical results were evaluated by two pathologists independently. Protein expression was assessed in a systematic fashion by cell counting 3 representative high-power field (x40 objective) per sample, approximately 100 to 200 cells/ fields. PD-L1 was considered positive when membranous tumor cell staining was observed in at least 1% of the tumor cells at any intensity according to the KEYNOTE- 010 study 14 (Fig 1). We also recorded the proportion of cells stained positive in all tumor area. Mean value among each subtype group was analyzed as shown in Table 2. Immune cells showing positive membranous or cytoplasmic after either PD1, CD4 or CD8 staining were considered positive.

### Double Staining IHC

Paraffin-embedded sections were heated at 60°C for 30 minutes and cooled to room temperature. Sequentially staining was done for the CD8 and the CD4 antibody. Deparaffinization and rehydration of tissue was done with graded concentrations of ethanol, and distilled water. Antigen retrieval was performed using Tris- EDTA pH 9 (for both antibodies) using a steamer at 120°C for 50 minutes and cooled to room temperature again. Sections were blocked with peroxidase blocking (Vector Laboratories, Burlingame, CA) for 1 hour at room temperature. Primary antibody application was performed overnight at 4°C. First, the primary antibody used was a Mouse Anti-CD8 (C8 / 468 + C8 / 144B Abcam at 1:500dil) and the next day slides incubated with 2nd polymer-HRP (DAKO EnVision™+ System, HRP) and visualized with DAB. For the secondary antibody, sections were incubated with Rabbit Anti-CD4 (EPR6855; Abcam, at 1:500dil) followed by avidin-biotin complex for 15 minutes at room temperature and developed alkaline phosphatase using a kit from Vector Laboratories. Finally, slides were counterstained with hematoxylin solution, dehydrated, and mounted.

### Immunofluorescense validation of antibodies and multiplexed immunofluorescense

After the chromogen-based IHC analysis was done, serial formalin-fixed, paraffin-embedded (FFPE) tissue with sections of 4-um thickness were used for monoplex immunoflourescence (IF) assay to optimize each antibody and to generate spectral libraries required for multiplex IF image analysis. Monoplex IF staining was performed manually by using the Opal 7 kit (catalogue # NEL797001KT; PerkinElmer, Waltham, MA), which uses individual tyramide signal amplification (TSA) conjugated fluorophores to detect various targets within an IF assay. After deparaffinization, slides were placed in a plastic container filled with antigen retrieval (AR) buffer in Tris-EDTA buffer pH 9.0 (PD-L1 and CD8 analysis) or citrate buffer pH 6.0 (for CD4 and PD-1 analysis); microwave technology was used to bring the liquid to the boiling point 1 min at 100 °C, and the sections were then microwaved for an additional 15 min at low power. Slides were allowed to cool in the AR buffer at room temperature and were then rinsed with deionized water and 1 × Tris-buffered saline with Tween 20 (TBST; Santa Cruz Biotechnology, Dallas, TX). To initiate protein stabilization and background reduction, Tris-HCl buffer containing 0.1% Tween (Dako, catalogue #S3022) was used for 10 min at room temperature. Slides were then incubated 1 hour with the primary antibodies, anti-PD-1 clone EH33 (1/400, Cell Signaling), anti-PD-L1 (1/200, Cell Signaling), anti-CD8 clone C8/468 + (1/100, Abcam, Cambridge; MA), and anti-CD4 EPR6855 (1/1000, Abcam; Cambridge; MA). Next, the slides were washed and incubated for 10 min at room temperature with anti-mouse or anti-rabbit secondary antibodies (Vector labs, Burlingame CA). The slides were then incubated at room temperature for 10 min with one of the following: Opal 540 (PD-L1), Opal 520 (CD4), Opal 570 (CD8) and Opal 650 (PD1), 1:50 dilution After additional washes in deionized water, the slides were counterstained with DAPI for 5 min and mounted with VECTASHIELD Hard Set (Vector Labs, Burlingame, CA). Similar to IHC validation, positive and negative controls were used during each staining run: human mature placenta for PDL-1, normal lymph node for CD4 and CD8 and human tonsil for PD-1.

Multiplex IF staining was done once each target was optimized in monoplex slides using the Opal 7 multiplexed assay. Staining was performed consecutively by using the same steps as those used in monoplex IF, and the detection for each marker was completed before application of the next antibody.

### Image collection and analysis

Images were acquired using a Zeiss AxioObserver Z1 widefield microscope (Carl Zeiss Microscopy, LLC, Thornwood, NY) equipped with 10x plan-apochromat (N.A. 0.45) objective lens, an Axioscam MRc5 color CCD camera for brightfield imaging, a Hamamatsu ORCA Flash 4 v2 sCMOS camera for fluorescence imaging, a CoolLED pE-4000 multi-LED fluorescence excitation light source, and Zen Blue (v2.3) image acquisition and processing software. Brightness and contrast in fluorescence images was adjusted by linear histogram stretching; the same adjustments were made for each image in the dataset. Images were exported as TIFF files and arranged into figures using Adobe Photoshop CC 2017.

### Statistical analysis

Descriptive analyses were calculated to describe the data population**.** Statistical analyses were performed using SPSS 15.0 (SPSS, Chicago IL USA).

## Results

### Clinicopathologic characteristics

The patient's characteristics is summarized in Table 1. One hundred and two patients were men, 70 were women. The median age of the patients at diagnosis was 48 years (range: 11-81 years old). Most of the samples included were kidney primary lesions (165) and seven were from distant metastasis of RCC including; samples from occipital dura, iliac bone, nasopharyngeal mass and neck lymph node from 4 HLRCC cases and 1 lung Metastasis (MT) from a CCRCC, 1 lung MT from a Type 1 PRCC and 1 Omentum sample from a SDHB mutated RCC. Radical nephrectomy was the most common type of surgery (90 cases), 51 had partial nephrectomy, 25 were excisional biopsies and 6 were incisional ones. Of the 165 kidney specimens the left side was affected in 91 cases, the right in 68 cases, 6 cases had bilateral tumors. Sixty patients had metastatic spread to one or more sites, (lymph nodes, lung and liver).

In our study, 59 (34 %) of the 172 tumors were positive for PD-L1 (Table 2) (Figure 1). Positive PD-L1 was found in the majority of HLRCC subtype (31/53, 58.49% cases), Type 1 Papillary RCC (10/31, 32.2% cases), chromophobe RCC (7/20, 35% cases), hybrid tumors (3/9, 33.3% cases), TFE3 tumors (3/8, 37.5%), undifferentiated (3/5, 60%) and TFEB tumors (2/2, 100%). (Table 2).

The number of positive intratumoral cells for PD-L1 in each group varied with the tumor type; in TFE3 the mean was 56.7%; TFEB was 55%; Type 1 PRCC was 41.1% (range 15-80); undifferentiated was 38.3%; HLRCC cases was 34.5%; hybrid tumors was 30% and chromophobe was 25.7% .

Cases of clear renal cell carcinoma, Oncocytomas and SDHB mutated tumors were all negative for PD-L1.

### PD-L1 and its relationship with PD-1, CD4 and CD8 lymphocytes

**Figure 2:**
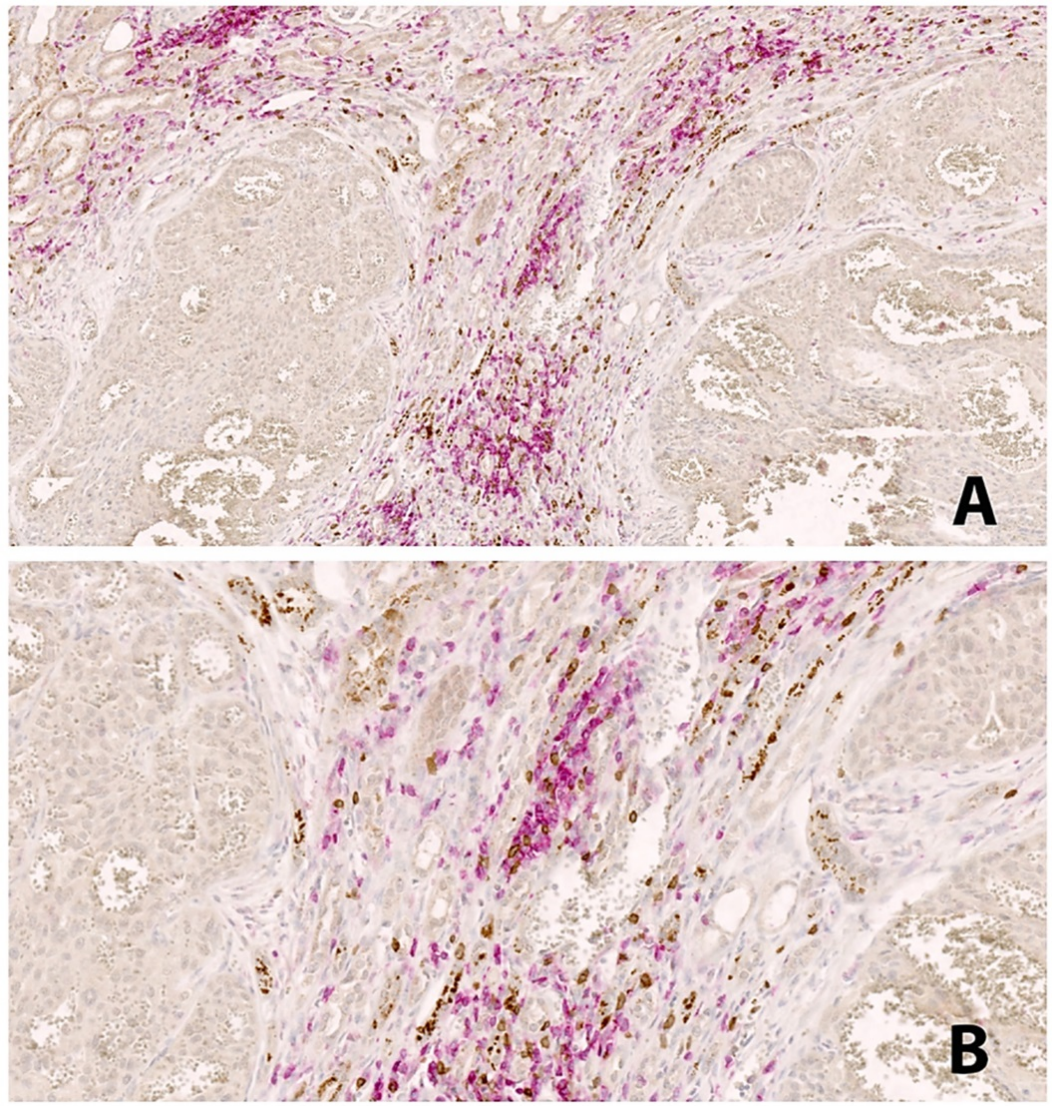
Double IHC staining in HLRCC. CD4^+^ lymphocytes in red color, CD8^+^ lymphocytes in brown color. The peritumoral distribution of immune cells is seen, with not immune cells within the tumor areas. Magnification: A: 20x, B:40x.

**Figure 3:**
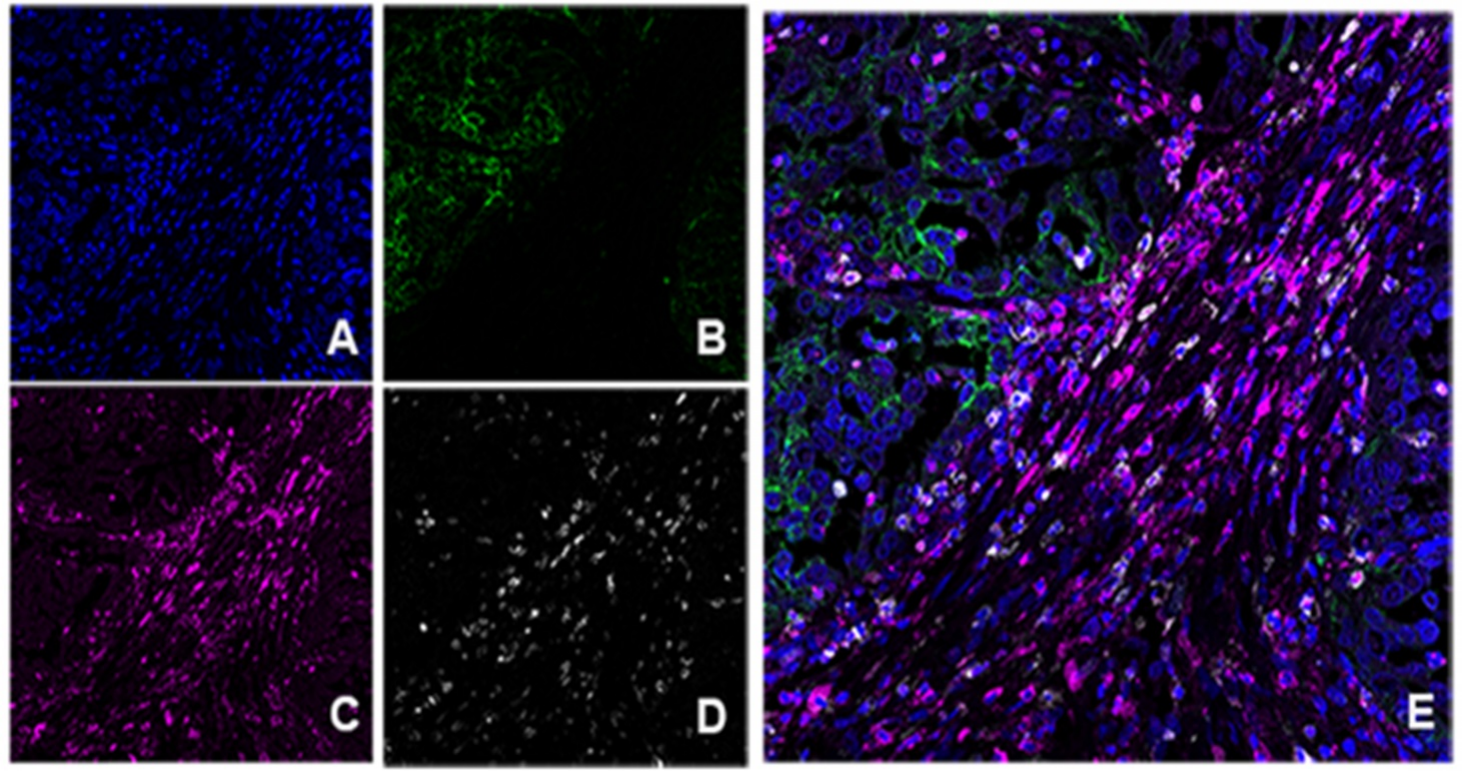
Multiplex immunofluorescence in HLRCC type. A. DAPI nuclear stain. B. PD-L1 positive in green color. C. PD-1 positive in magenta. D. CD8^+^ in white. E. Combination of markers without unmixing colors; note positive staining of PD-L1 at the tumor area (green) and peritumoral distribution of cells co-expressing CD8^+^ and PD1^+^ (pink color: magenta and white overlapped).

In our Multiplex IF analysis, PD-L1 expression was present mainly on tumor cells and macrophages, while PD-1 was expressed on CD4, and CD8 T cells. HLRCC tumors, which are associated with high intratumoral PD-L1 and lymphocytes PD-1 expression, had the most pronounced inflammatory infiltrate of both CD4 and CD8 T cells, particularly the latter. The location of the T cells is also interesting in this subtype of tumor, with cells surrounding the tumor, and very rarely intraepithelial (Figure 2). Co-expression of PD-1 and CD8 was demonstrated by co-localization of the two signals on lymphocytes present within the periphery of the tumor (Figure 3).

In contrast, in the subgroup of clear cell carcinomas, which were negative for PD-L1, there was a sparse T cell infiltrate in comparison with the renal carcinoma subtypes that had PD-L1 expression.

## Discussion

Previous studies reported that high PD-L1 expression was regarded as a poor prognostic biomarker in patients with lung cancer, breast cancer, malignant melanoma, hepatocellular, gastric, pancreatic, and ovarian cancers 15-17. The present study demonstrates that in kidney cancer high PD-L1 expression is seen in aggressive forms such as HLRCC as well as other types like papillary type 1, chromophobe, hybrid and MiT family Translocation tumors. Choueiri, et al. observed similar results, when they performed an exploratory multivariate analysis that showed that the PD-L1 expression in non- clear cell RCC is heterogeneous, and depends upon tumor stage and histology, being significant effect modifiers for the association of PD-L1 positivity on clinical outcome 18, 19.

Reported studies of PD-L1 expression were associated with poor prognostic histological factors, such as tumor stage, ISUP nucleolar grade, and sarcomatoid component 20. In our study the highest proportion of positive cases were HLRCC. This entity is a hereditary cancer syndrome in which affected individuals are predisposed to the development of leiomyomas of the skin and uterus 21, as well as an aggressive kidney cancer 22. The disease is inherited as an autosomal dominant condition with incomplete phenotype penetrance, and germline mutations in the fumarate hydratase (FH, 1q42.3-q43) gene 23, 24. Patients with HLRCC frequently present at an advanced stage with lymph node metastases.

In contrast, the clear cells RCC subtype had no expression for PD-L1. Previous publications reported that sporadic clear cell RCC were particularly associated with PD-L1. These results support the theory of alternative oncogenic pathways in clear cell RCC, leading to PD-L1 overexpression, despite hypoxia-inducible factor degradation due to the presence of an activated VHL protein. Tumors with no inactivation of VHL can perhaps use alternative pathways independent of VHL mechanisms, such as the MAP kinase and PI3K- AKT-mTOR pathways involved in clear cell RCC oncogenesis. these alternative pathways have already been reported to induce PD-L1 expression in responses in other cancers 25.

In HLRCC increased PD-L1 expression is associated with increased numbers of CD8 TILs expressing PD-1 in the tumor margins. It is known that PD-1 can be expressed on numerous cell types; in addition to CD8 T cells, it is also expressed in CD4 cells; as our stained tissues show, and previous papers demonstrated that it can be expressed in B cells 26. PD-L1 expression has also been reported in dendritic cells, macrophages, and plasma cells 26, 27.

Our results showed that increased expression of PD-L1, PD-1, and CD8 are associated with one another. PD-L1 can be either constitutively expressed or induced via localized inflammatory stimuli within the tumor microenvironment, such as interferons 26-29. It may be that a subset of tumors has preexisting constitutive expression of PD-L1, in addition to induction by inflammatory stimuli. Alternatively, HLRCC may represent a subset of RCC that are more responsive to inflammatory stimuli, resulting in PD-L1 induction.

Expression patterns of PD-L1 in close proximity to PD-1-positive CD8 TILs at the tumoral margin are similar to those previously reported in melanoma and some sarcomas 30.

RCCs shows that the expression of PD-L1 is associated with the histological subtype, and over expression of PD-L1 could be a predictor of poor prognosis. Modern immunotherapy, specifically immune checkpoint inhibitors such as anti-programmed death receptor 1 (anti-PD-1) and anti-programmed death receptor ligand 1 (anti-PD-L1) antibodies may well be an important new modality in the treatment of kidney cancer. One of these drugs, the anti-PD-1 antibody nivolumab, was FDA-approved for kidney cancer in 2015. Now there is significant interest in evaluating combination immunotherapy strategies including (1) PD-1/PD-L1 inhibition plus other checkpoint inhibitors, T cell agonists or microenvironment modifying agents, (2) PD-1/PDL1 inhibition plus (personalized) vaccination approaches, (3) PD-1/PD-L1 inhibition plus adoptive T cell therapy.

Therefore, evaluation of PD-L1 and PD-1 in RCCs tissue samples is and will be important to predict immunotherapy potential, which will likely dominate therapeutic approaches in the future.

## Figures and Tables

**Table 1:** Clinicopathologic Findings

Variables	Number of Patients (n:172)
**Gender**	
Male	102
Female	70
**Age (Years)**	48 (11-81)
**Site**	
Kidney	165
Distant metastasis	7
**Laterality (kidney tumors)**	
Right	68
Left	91
Bilateral	6

**Table 2:** Positive cases for PDL1 according to subtype of RCCs

Subtype of RCC	Number of Patients (N:172)	Positive Cases for PDL1	Mean Positivity of PD-L1 in Tumor Cells
HLRCC	53	31 (58.4%)	34.5%
Clear Cell RCC (VHL)	34	0 (0%)	0 %
Type 1 PRCC	31	10 (32.2%)	41.1%
Chromophobe RCC	20	7 (35.0%)	25.7%
Hybrid Tumor	9	3 (33.3%)	30%
RCC-TFE3	8	3 (37.5%)	56.7%
Oncocytoma	7	0 (0%)	0 %
Undifferentiated	5	3 (60%)	38.3%
RCC with SDHB mutation	3	0 (0%)	0%
TFEB-RCC	2	2 (100%)	55%
**Total**	172	59	
